# The dorsal vagal complex harbors a constitutively immune-enriched microglial population

**DOI:** 10.3389/fnana.2026.1923233

**Published:** 2026-10-21

**Authors:** Stéphanie Gaige, Rym Barbouche, Alain Tonetto, Jean-Denis Troadec, Anne Abysique

**Affiliations:** 1 Centre de Recherche en Psychologie et Neurosciences (CRPN), Aix-Marseille University, CNRS UMR 7077, Marseille, France; 2 Plateforme de Recherche Analytique Technologique et Imagerie (PRATIM), FSCM (UAR1739), CNRS, Centrale Marseille, Aix-Marseille University, Marseille, France

**Keywords:** brainstem, Fc receptors, healthy brain, IgG, MHC I, MHC II, microglia, NTS

## Abstract

The dorsal vagal complex (DVC) comprises three anatomically and functionally linked structures, the area postrema, the nucleus tractus solitarius, and the dorsal motor nucleus of the vagus nerve, forming a key hub for the integration of peripheral inflammatory information. These structures play a unique role at the neuroimmune interface, conveying peripheral immune signals to the brain and generating behavioral and physiological responses through two complementary pathways: a neural pathway, *via* vagal afferents projecting to the nucleus tractus solitarius, and a humoral pathway, *via* the area postrema, which lacks a blood–brain barrier and is therefore sensitive to circulating inflammatory mediators. Numerous studies have established that the DVC serves as a mandatory gateway for peripheral inflammatory signals, integrating this information prior to its transmission to forebrain centers and peripheral organs. Understanding this interface represents a major therapeutic challenge, as modulating the DVC and its circuits opens new avenues for the treatment of chronic inflammatory diseases and neurodegenerative disorders in which this interface is chronically disrupted. Paradoxically, despite this essential role in the regulation of inflammation, the phenotype of microglial cells, the resident immune cells of the central nervous system, has received very little attention in this region. In the present study, we characterized these cells by investigating the expression of Fc receptors for IgG and major histocompatibility complex class I and II. Our results reveal microglial subpopulations displaying an atypical phenotype in the healthy DVC, suggesting that these cells may serve as a specialized immunological interface capable of actively monitoring systemic signals.

## 1 Introduction

Located at the base of the fourth ventricle, the DVC comprises three anatomically and functionally interconnected structures of the medullary brainstem, the area postrema (AP), the nucleus tractus solitarius (NTS), and the dorsal motor nucleus of the vagus nerve (DMNX), that together constitute a critical neuroimmune interface between the periphery and the brain. These structures play a unique role in conveying peripheral immune information to the rest of the brain and in generating behavioral and physiological responses (Marvel et al., 2004). The AP is a circumventricular organ that lacks a functional blood–brain barrier (BBB). Its fenestrated endothelial capillaries allow circulating mediators, including cytokines, hormones, and bacterial toxins, to directly access AP cells without requiring transcellular transport, thus enabling real-time detection of systemic inflammation via membrane receptors expressed on the surface of its neurons and astrocytes (van de Lisdonk and Li, 2024). The mandatory role of the AP as a transducer of circulating immune signals to forebrain structures has been demonstrated by the fact that its surgical ablation completely abolishes the hypothalamic–pituitary–adrenal (HPA) axis response induced by intravenous IL-1β administration, without altering peripheral cytokine levels (Lee et al., 1998). In parallel with this slow humoral pathway, vagal afferents constitute a rapid neural pathway for immune-to-brain communication. Sensory neurons of the nodose ganglion constitutively express IL-1R1, TNFR1, and TLR4 (Hosoi et al., 2005), enabling direct detection of circulating inflammatory mediators without crossing the BBB (Huerta et al., 2025). The functional relevance of this pathway has been established by two converging sets of experimental evidence: subdiaphragmatic vagotomy abolishes LPS-induced IL-1β mRNA expression in the brain without affecting peripheral cytokine levels (Layé et al., 1995), and similarly blocks the illness behavior induced by intraperitoneal, but not intravenous, IL-1b administration (Bluthé et al., 1996). These results collectively demonstrate that vagal afferents are specifically required for the detection of low-intensity intra-abdominal immune signals, operating below the threshold needed to activate the humoral pathway. The NTS thus receives a convergence of visceral afferent information from the gastrointestinal, cardiovascular, respiratory, and immune systems, serving as the primary site of integration for these signals. It subsequently contributes to the dissemination of inflammatory information *via* direct catecholaminergic projections to key forebrain structures, including the paraventricular nucleus of the hypothalamus, the central amygdala, and the bed nucleus of the stria terminalis. It is important to note that, electrical stimulation of vagal afferents is sufficient to induce hypothalamic IL-1β and CRF expression and to elevate plasma ACTH and corticosterone levels, thus confirming the NTS as a central orchestrator of the neuroendocrine response to peripheral inflammation (Hosoi et al., 2000). Beyond its role as a passive relay, the DVC generates a regulatory efferent response *via* the DMNX, forming the complete arc of the inflammatory reflex conceptualized by Tracey (2002). This homeostatic circuit enables real-time regulation of peripheral inflammation by the brain through the cholinergic anti-inflammatory pathway, whose molecular and cellular components have been progressively elucidated (Rosas-Ballina et al., 2011) and whose therapeutic relevance has been recently demonstrated in a pivotal randomized controlled trial in rheumatoid arthritis (Tesser et al., 2026).

Microglial cells, distributed throughout the entire central nervous system (CNS), are the resident immune cells of the brain and play a pivotal role in homeostatic surveillance and the cerebral inflammatory response (Gaige et al., 2022). As immune sentinels, they actively monitor the brain’s microenvironment, participate in innate and adaptive immune responses, and protect the brain from injury and infection. Given the essential role of the DVC in the bidirectional integration of immune information between the periphery and the brain, the characterization of its microglial population represents a crucial step toward understanding immune information processing under both physiological and pathological conditions. Recent studies have begun to reveal microglial populations exhibiting an atypical phenotype within this structure (Takagi et al., 2019; Gaige et al., 2022). Indeed, subpopulations of microglial cells residing in the NTS and around the AP are maintained in a functionally activated state even under physiological conditions, and it has been suggested that these populations serve to protect the brain parenchyma from hazardous blood-borne molecules (Gaige et al., 2022).

In this context, the present study aimed to deepen our understanding of DVC microglia by characterizing the potential expression of two families of immunologically relevant surface molecules: Fcγ receptors (FcγR), a membrane receptors that bind to the Fc fragment of circulating IgG or immune complexes (ICs) and mediate phagocytosis, cytotoxicity, and immune activation, and major histocompatibility complex (MHC) class I and II molecules, which present peptide fragments derived from pathogens or endogenous proteins to cytotoxic CD8^+^ and CD4^+^ T lymphocytes, respectively, thereby bridging innate and adaptive immunity within the CNS.

## 2 Materials and methods

### 2.1 Animal, diets, and longitudinal monitoring

Adult male C57BL/6J mice were obtained from Charles River Laboratories (L’Abresle, France). They were housed in standard cages under controlled environmental conditions, including a 12-h light–dark cycle (7:00 a.m. to 7:00 p.m.) and an ambient temperature of 22 °C. Mice were housed in groups of 3 or 4 with free access to water and normal chow pellets (SAFE, Augy, France).

### 2.2 Immunohistochemistry

Mice were given buprenorphine subcutaneously (0.05 mg/kg) for analgesia before being anesthetized by intraperitoneal injection of ketamine (120 mg/kg, Imalgène 1,000, Boehringer Ingelheim) and xylazine (16 mg/kg, Rompu 2, Bayer Santé). The perfusion procedure consisted of administering 2.5 mL of 0.1 M phosphate buffer saline (PBS) at pH 7.4 containing 10% heparin (Choay, Cheplapharm France), followed by cold 0.1 M PBS, and then cold 4% paraformaldehyde (PFA) in 0.1 M PBS. The brains were immediately removed, post-fixed in 4% PFA for 1 h at room temperature, and then rinsed overnight in PBS. They were cryoprotected for 24–48 h at 4 °C in a 30% sucrose solution prepared in PBS. The brains were then frozen by immersion in isopentane at −40 °C, and coronal sections (40 μm thick) containing the DVC were obtained using a cryostat (Leica CM3050, France) and collected serially in 0.1 M PBS. Some tissue sections were preserved in an antifreeze solution (Ethylene glycol and glycerol based cryoprotectant) before proceeding with the immunohistochemistry protocol. Floating-section immunostaining was performed for the different immunolabeling. Sections were incubated for 1 h in a blocking solution and then processed for immunostaining. The conditions for double immunostaining including antibody references, working dilution and saturation solution were summarized in Table 1. All antibodies were applied at room temperature, overnight for primary antibodies and for 90 min for secondary antibodies. After washing in PBS, sections were mounted on gelatin-coated slides and coverslipped with Mowiol mounting medium.

**Table 1:** Immunohistochemistry conditions.

Immunostaining	Primary antibodies	Secondary antibodies	Blocking
IgG/IBA-1	-No primary antibody -Goat polyclonal anti-IBA1 (1:500) ab5076, Abcam	-Donkey anti-Mouse Alexa Fluor 594 (1:400), A21203, Invitrogen -Donkey anti-Goat Alexa Fluor 488 (1:400) A11055, Invitrogen	-Donkey serum 3% -Horse serum 3%
IBA-1/CD68	-Goat polyclonal anti-IBA1 (1:500) ab5076, Abcam -Mouse monoclonal anti-CD68 (1: 200) E-AB-22013, Elabscience	-Donkey anti-Goat Alexa Fluor 594 (1:400) A11058, Invitrogen -Goat anti-Mouse Alexa Fluor 488 (1:400) A11029, Invitrogen	-Horse serum 3% -BSA 3%
IgG/CD11b	-No primary antibody -Rat monoclonal anti-CD11b (1: 100) Sc-23937, Santa Cruz Biotechnology	Donkey anti-Mouse Alexa Fluor 594 (1:400), A21203, Invitrogen -Goat anti-Rat Alexa Fluor 488 (1:400) A11006, Invitrogen	-Donkey serum 3% -BSA 3%
IBA-1/CD64	-Goat polyclonal anti-IBA1 (1:500) ab5076, Abcam -Mouse monoclonal anti-CD64 (1:200) Sc-1184, Santa Cruz Biotechnology	-Donkey anti-Goat Alexa Fluor 488 (1:400) (A11055, Invitrogen) -Goat anti-Mouse Alexa Fluor 594 (1:400) A11032, Invitrogen	-Horse serum 3% -BSA 3%
IBA-1/MHC 1	-Goat polyclonal anti-IBA1 (1:500) ab5076, Abcam -Mouse monoclonal anti-CMH 1/HLA class I (1:200), Sc-69892, Santa Cruz Biotechnology	-Donkey anti-Goat Alexa Fluor 488 (1:400) A11055, Invitrogen -Goat anti-Mouse Alexa Fluor 594 (1:400) A11032, Invitrogen	-Horse serum 3% -Normal Goat serum 3%
IBA-1/MHC 2	-Goat polyclonal anti-IBA1 (1:500) ab5076, Abcam -Mouse monoclonal anti-CMH 2/HLA-DR (1:200), Sc-53319, Santa Cruz Biotechnology	-Donkey anti-Goat Alexa Fluor 488 (1:400) A11055, Invitrogen -Goat anti-Mouse Alexa Fluor 594 (1:400) A11032, Invitrogen	-Horse serum 3% -Normal Goat serum 3%
FcRn/CD206	-Mouse monoclonal anti-FcRn (1:200) Sc-393064, Santa Cruz Biotechnology -Goat polyclonal anti-CD206 (1:500) AF2535, R&D Systems	-Goat anti-Mouse Alexa Fluor 594 (1:400) A11032, Invitrogen -Donkey anti-Goat Alexa Fluor 488 (1:400) A11055, Invitrogen	-BSA 3% -BSA 3%
IBA-1/FcRn	-Goat polyclonal anti-IBA1 (1:500) ab5076, Abcam -Mouse monoclonal anti-FcRn (1:200) Sc-393064, Santa Cruz Biotechnology	-Donkey anti-Goat Alexa Fluor 488 (1:400) A11055, Invitrogen -Goat anti-Mouse Alexa Fluor 594 (1:400) A11032, Invitrogen	-Horse serum 3% -BSA 3%
IBA-1/CD16/32	-Goat polyclonal anti-IBA1 (1:500) ab5076, Abcam -Rat monoclonal anti-CD16/32 (1:200) Orb1293362, Biorbyt LTD	-Donkey anti-Goat Alexa Fluor 488 (1:400) A11055, Invitrogen -Goat anti-Rat Alexa Fluor 564 (1:400) A11007, Invitrogen	-Horse serum 3% -Horse serum 3%
TMEM119/IBA1	-Rabbit polyclonal anti-TMEM119 (1:2000) GTX134087, GeneTex -Goat polyclonal anti-IBA1 (1:500) ab5076, Abcam	Donkey anti-Rabbit Alexa Fluor 594 (1:400) A21207, Invitrogen -Donkey anti-Goat Alexa Fluor 488 (1:400) A11055, Invitrogen	-BSA 3% -Horse serum 3%

It is worth mentioning that while MHC class I and class II antibodies were raised against human antigens, they are manufacturer-recommended for murine applications. The use of these human-raised antibodies in murine tissue is justified by literature data. Indeed, a high sequence homology exists between human and murine MHC class I (Malissen et al., 1982), while the MHC class II antibody has been experimentally validated on murine cells (Loi et al., 2015; Sun et al., 2025).

### 2.3 Image acquisition and data processing

The fluorescence images were acquired using a Zeiss LSM 710 confocal microscope and Zen software. For double-labeling experiments, the images were acquired sequentially. In some cases, mosaics of images at x20 magnification were created to visualize the entire section with high definition. All images were processed with Adobe Photoshop CS6, limited to contrast and brightness adjustments. To quantify the intensity of each labeling in different subareas of the brainstem, confocal fluorescent images were acquired in Z-stack mode to capture the complete cell arborization. Laser and detector settings were kept constant for all images to ensure comparability between animals. A maximum intensity projection of different focal planes was then generated to create a single image. To evaluate labeling intensity, analyses were performed using ImageJ Fiji 1.54f software (NIH, Bethesda, MD, United States). Images were converted to grayscale, inverted, and binarized (threshold: *method*) using ImageJ Fiji 1.54f to depict IBA1^+^ cells and their processes in black on a white background. Background was subtracted and thresholding was applied. The average grey level intensity of each labeling in each subregion of interest was quantified using the “Analyze” function in ImageJ Fiji 1.54f. Given the marked heterogeneity of labeling within the NTS, we chose to conduct an analysis of NTS subnuclei. Several authors have proposed delineations of the various NTS subnuclei, often based on cytoarchitectonic parcellation (Ganchrow et al., 2014; Holt, 2022). We relied on these studies to define our regions of interest, grouping subnuclei together to simplify the analysis. The term commissural/postremal NTS (cpNTS) refers to the grouping of the commissural subnucleus, the parvocellular subnucleus and the *funiculus separans*, the term centromedial NTS (cmNTS) groups the medial, dorsomedial and central subnuclei and the term lateral NTS (lNTS) encompasses the ventral, ventrolateral and dorsolateral subnuclei. For each quantified staining, the analysis was performed on three non-consecutive sections of postremal DVC per animal. The average gray intensity was calculated based on a sample of 4 to 8 animals for each immunostaining.

### 2.4 Statistical analysis

All results are presented as mean ± SEM. Statistical analysis was performed using GraphPad Prism version 6.0 (GraphPad Software, San Diego, CA, United States). Immunofluorescence labeling intensity was compared between regions using one-way ANOVA followed by Tukey’s *post hoc* test. Statistical significance was set at *p* < 0.05. In the figures, different letters indicate statistically significant differences, whereas groups sharing the same letter do not differ significantly.

## 3 Results

### 3.1 Fc receptor and FcRn expression in the DVC

We first examined the expression of Fc receptors to IgG (FcγRs) and neonatal Fc receptor (FcRn) in the DVC (Figure 1). In mice, FcγRs bind IgG with varying affinities and are subdivided into three classes: FcγRI (CD64; a high-affinity receptor for monomeric IgG), FcγRIIb (CD32, an inhibitory fc receptor), and FcγRIII (CD16; a low-affinity receptor for ICs). For all markers examined, immunoreactivity was most pronounced in the postremal region of the DVC, with labeling intensity decreasing markedly in the caudal and rostral portions of the structure; the following analysis is therefore restricted to this region. CD16/32 (FcγRIII/FcγRIIb) immunostaining revealed a relatively uniform labeling pattern throughout the DVC and in adjacent structures, including the hypoglossal nucleus (Figures 1A,O,P). Double labeling with the microglial marker IBA1 demonstrated that all CD16/32-immunoreactive cells were microglia, with virtually all IBA1^+^ cells co-expressing CD16/32 (Figures 1B,C). CD64 (FcγRI) immunostaining revealed an intense and spatially restricted signal at the AP/NTS border corresponding to the *funiculus separans,* the commissural NTS and the parvocellular NTS (grouped here under the term cpNTS) which gradually faded toward the lateral parts of the NTS namely, centromedial (cm) and lateral (l) NTS (Figures 1D,O,P). The labeling was markedly weaker in the AP than in the commissural/postremal NTS. A strong colocalization of CD64 with IBA1 was observed in the NTS and the AP (Figures 1E,F), identifying microglial cells as the main cellular population expressing CD64 in these subregions. In the AP and cpNTS CD64 immunoreactivity was also detected in IBA1^−^ structures consistent with blood vessels.

**Figure 1:**
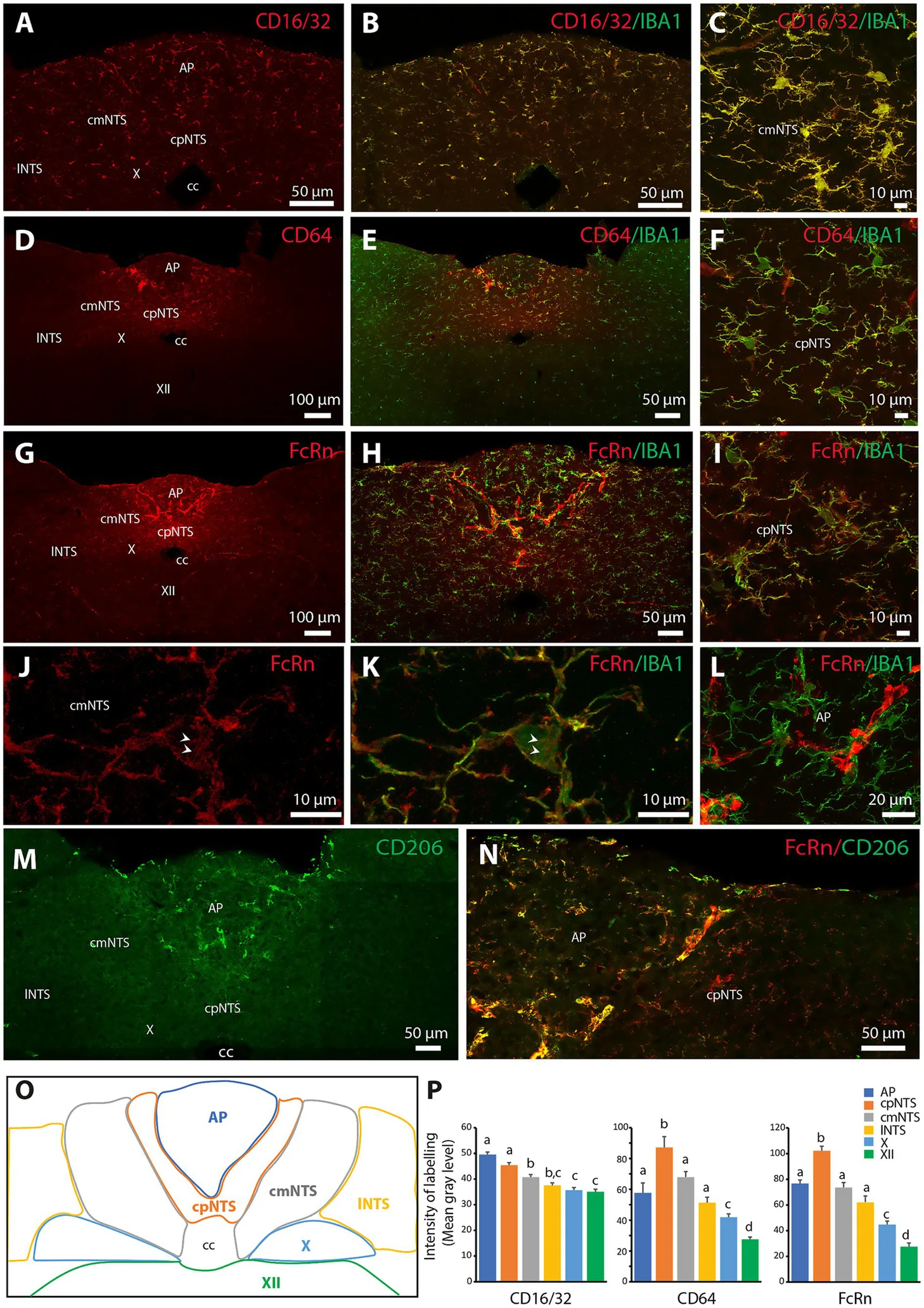
Differential expression of IgG Fc receptors (FcγRs) and neonatal Fc receptor (FcRn) by microglial cells in the DVC. **(A–I)** Representative confocal images (maximal projection) at increasing magnifications illustrating the immunolabeling of CD16/32 [FcγRIII/FcγRIIb; **(A–C)**], CD64 [FcγRI; **(D–F)**], and FcRn **(G–I)** in the DVC, co-labeled with IBA1 (microglial marker). **(J,K)** High-magnification images (0.4 μm optical sections) for FcRn and IBA1 illustrate the punctiform FcRn labeling (arrowheads) observed in the cytosol of microglial located in the dorsomedial NTS. **(L)** Double immunostaining for FcRn and IBA1 demonstrating the mutually exclusive expression of these two receptors by distinct cell populations within the AP. **(M)** Labeling of CD206, a marker of alternatively activated macrophages, in the DVC highlighting a labeling strongly associated with the AP. **(N)** Representative confocal images of CD206 and FcRn co-labeling showing colocalization of the two markers in the AP. **(O)** Diagram showing the boundaries of the AP, X and NTS subdivisions used to quantify the labeling intensity of the various Fc receptors in the different parts of the DVC. **(P)** Histograms showing the intensity of Fc receptors labeling in DVC subregions and hypoglossal nucleus (*n* = 8 for each labeling). Different letters indicate statistically significant differences (*p* < 0.05). AP, area postrema; cc, central canal; cpNTS, commissural/postremal nucleus tractus solitarius; cmNTS, centromedial NTS; lNTS, lateral NTS; X, dorsal motor nucleus of the vagus nerve; XII, hypoglossal nucleus. IBA1, Ionized Calcium-Binding Adaptor Molecule 1; FcRn, neonatal Fc receptor, CD206, macrophage mannose receptor.

We next searched for the possible FcRn expression in the DVC. Its spatial distribution closely mirrored that of CD64, with strong immunoreactivity at the cpNTS which gradually decreased toward the cmNTS and lNTS (Figures 1G,O,P). FcRn was strongly colocalized with IBA1 in the cpNTS (Figures 1I,K), where microglial cells displayed a characteristic punctate intracellular labeling distributed throughout the entire cellular arborization (Figures 1J–K). In contrast, in the AP, FcRn immunoreactivity was weakly associated with IBA1^+^ cells (Figure 1L) but rather colocalized with blood vessels and CD206^+^ cells (Figures 1M,N), a phenotypic profile consistent with perivascular macrophages. CD206, the macrophage mannose receptor, is a C-type lectin expressed predominantly by macrophages, dendritic cells and some endothelial cells. Notably, no FcγR or FcRn immunoreactivity was detected in DVC neurons for any of the markers examined.

### 3.2 IgG accumulation in the DVC

Having established that multiple FcγRs are highly expressed in the non-inflamed DVC by microglial cells, we next asked whether this receptor landscape was associated with high local IgG concentrations. IgG was detected using anti-mouse IgG (H + L) antibody conjugated to Alexa Fluor 594. Specific immunofluorescence revealed a significantly higher concentration of IgG in the DVC particularly within the AP, cpNTS and cmNTS compared to surrounding bulbar structures, i.e., hypoglossal nucleus thus reproducing the spatial gradient previously observed for CD64, and FcRn (Figures 2A–G). Co-staining with IBA1 (Figures 2H–J**)** or CD11b (Figures 2K–M) confirmed that almost all microglial cells in the DVC were IgG-positive, with labeling intensity again gradually decreasing towards the lateral NTS. CD11b is a microglial marker whose expression correlates to the severity of microglial activation (Rock et al., 2004). High-magnification imaging in 0.4 μm optical sections revealed IgG labeling at the plasma membrane of IBA1^+^ microglial cells, with additional intracellular signal detected in a subset of cells (Figures 2J,M), suggesting active endocytosis and intracellular processing of IgG. To confirm the microglial phenotype of IBA1^+^IgG^+^ cells observed in the DVC, we used TMEM119 as an additional microglial marker. Double labeling for TMEM119 and IBA1 revealed that, in all parts of the DVC, the vast majority of IBA1^+^ cells were strongly positive for TMEM119, except in the AP and the solitary tract, where IBA1^+^ cells expressed TMEM119 only weakly (Figures 2N,O).

**Figure 2:**
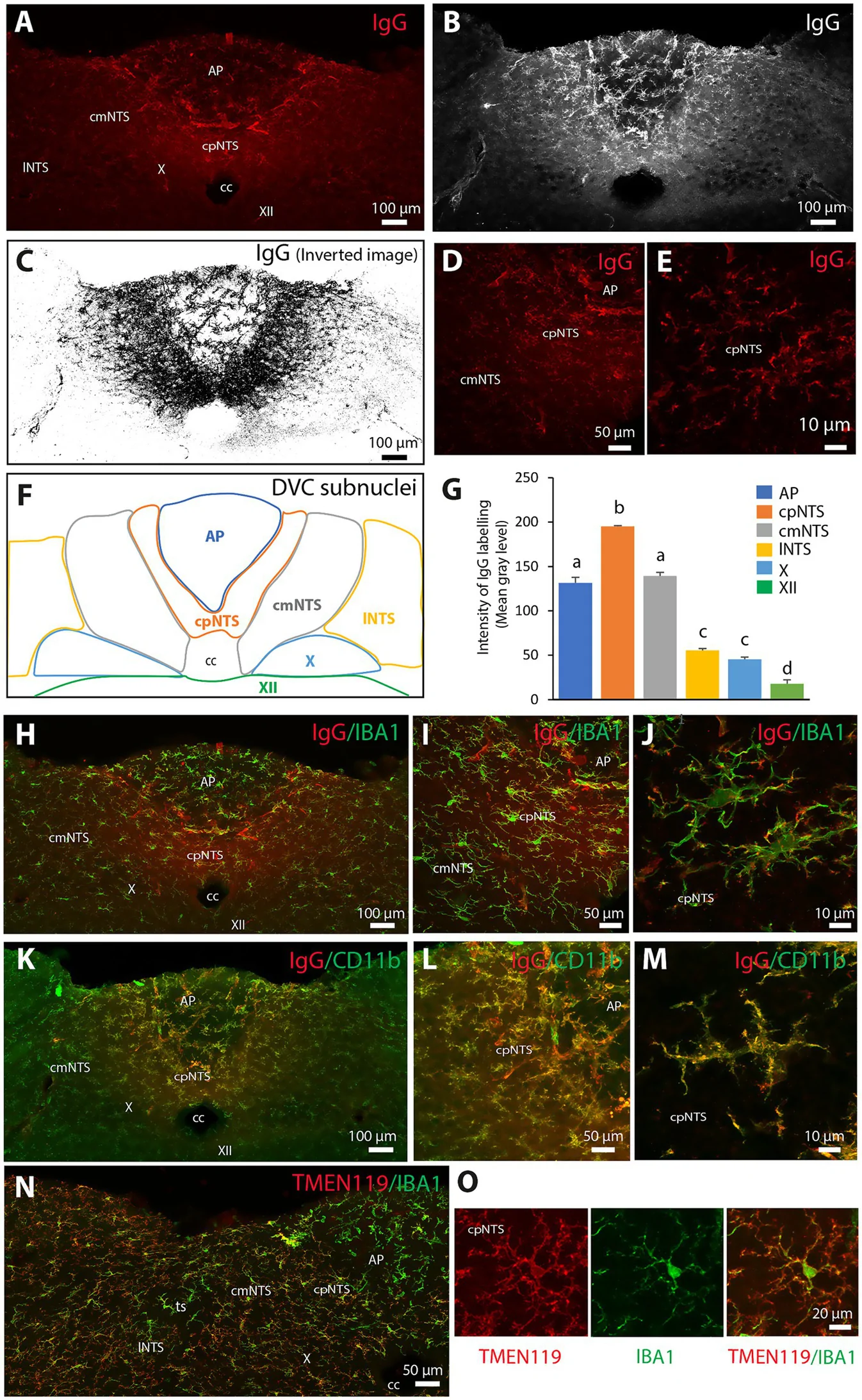
IgG accumulation in the DVC is associated with microglial cells. **(A–C)** Representative confocal images [**(A)**, red fluorescence; **(B)**, grey level image; and **(C)**, inverted image] of immunofluorescent IgG staining, with labeling intensity mirroring the spatial gradient observed for FcγR expression. **(D,E)** High-magnification images of IgG labeling illustrating the spatial gradient between the AP, cpNTS and cmNTS **(D)** and the cellular labeling of cells located in the cpNTS **(E)**. **(F)** Diagram showing the boundaries of the AP, X and NTS subdivisions used to quantify the labeling intensity of IgG in the different parts of the DVC. **(G)** Histogram showing the intensity of IgG labeling in DVC subregions and hypoglossal nucleus illustrating their spatial distribution (*n* = 8). Different letters indicate statistically significant differences (*p* < 0.05). **(H–M)** Representative confocal images of immunofluorescent co-staining for IgG and either IBA1 **(H–J)** or CD11b **(K,M)** in the DVC. IgG immunoreactivity strongly co-localized with both microglial markers throughout the DVC. High-magnification images (J for IBA1; M for CD11b) reveal IgG distribution throughout the entire microglial arborization, with labeling detected both at the plasma membrane and within the cytosol of IBA1^+^. **(N,O)** Representative confocal images (maximal projection) at different magnifications illustrating the co-labeling of TMEM119 and IBA1 in the DVC. AP, area postrema; cc, central canal; cpNTS, commissural/postremal nucleus tractus solitarius; cmNTS, centromedial NTS; lNTS, lateral NTS; X, dorsal motor nucleus of the vagus nerve; XII, hypoglossal nucleus. IBA1, Ionized Calcium-Binding Adaptor Molecule 1; CD11b, integrin alpha-M subunit (microglial marker). TMEN-119, Transmembrane protein 119; (*n* = 4 per co-labeling condition).

### 3.3 MHC class I and II expression in DVC microglia

Given the marked IgG association with DVC microglial cells, we investigated whether these cells also express MHC class I and II molecules, the key molecules of antigen presentation to T lymphocytes and the gateway to adaptive immunity (Van Meerhaeghe et al., 2023). Both molecules were detected in the DVC. MHC class I was highly expressed by microglial cells in the NTS and DMNX, with expression decreasing progressively with distance from the cpNTS (Figure 3). Membrane-level labeling predominated at the cellular scale (Figures 3E–G). MHC class II immunolabeling was visible in all parts of the DVC (Figures 3H,I), clearly delineating the DVC from adjacent structures such as the hypoglossal nucleus, which were poorly labelled for this marker (Figures 3H,I). As observed with Fc receptors and IgG, the intensity of MHC class I and II labeling reached its maximum in the cpNTS and gradually faded as the distance from the AP increased (Figures 3B,I). At the cellular level, MHC class II immunoreactivity was colocalized with IBA1^+^ microglial cells (Figures 3J–N) as well as with blood vessels, particularly in the AP (Figure 3K).

**Figure 3:**
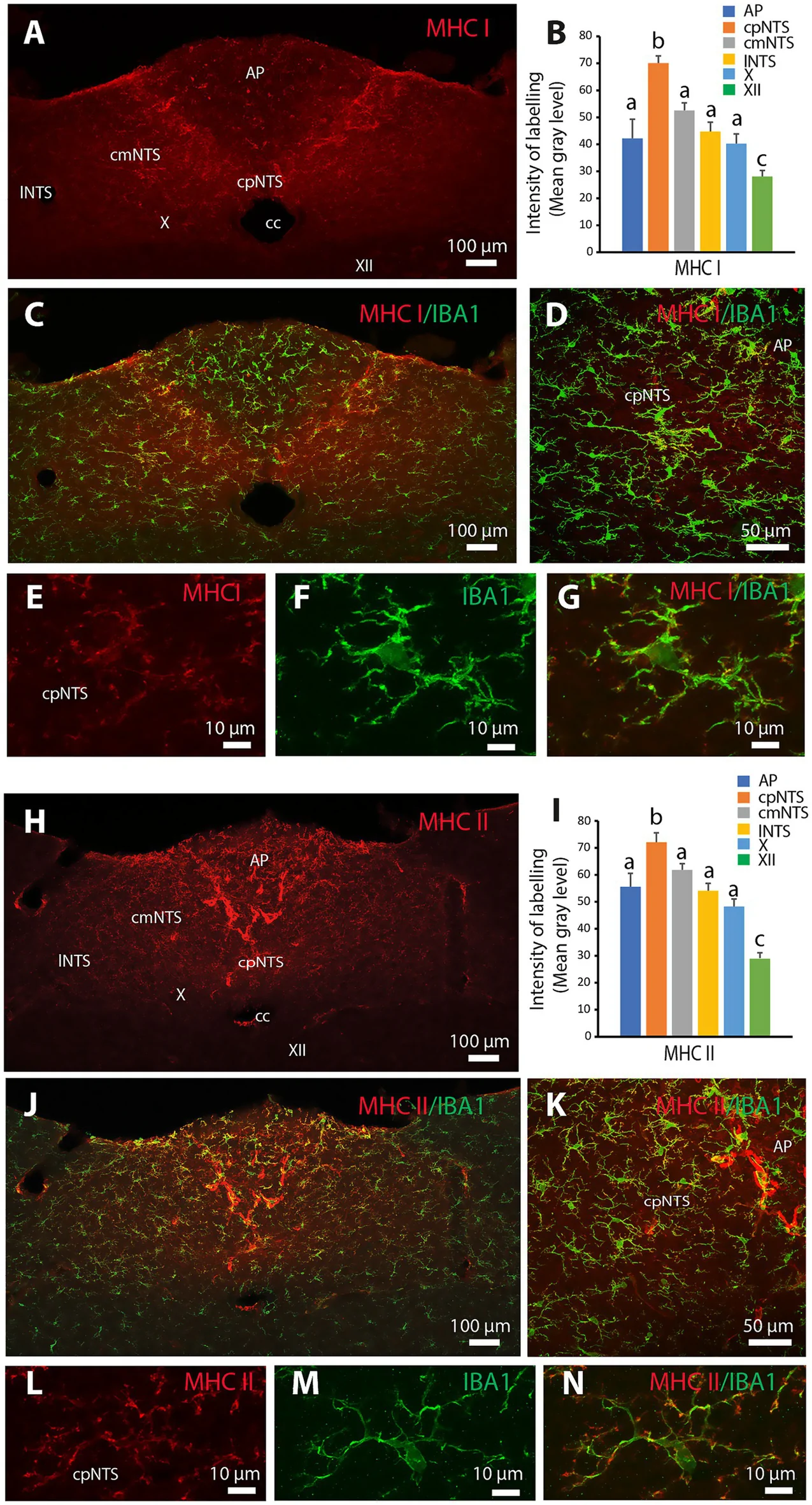
Constitutive expression of MHC class I and II molecules by microglial cells in the DVC. **(A)** Representative confocal image illustrating the immunolabeling of MHC class I in DVC. **(B)** Histogram showing the intensity of MHC class I labeling in DVC subregions and hypoglossal nucleus (*n* = 8 animals). Different letters indicate statistically significant differences (*p* < 0.05). **(C–G)** Representative confocal images (maximal projection) at increasing magnifications illustrating the immunolabeling of MHC class I in the DVC, co-labeled with IBA1 (microglial marker, green, *n* = 4). **(H)** Representative confocal image illustrating the immunolabeling of MHC class II in DVC. **(I)** Histogram showing the intensity of MHC class II labeling in DVC subregions and hypoglossal nucleus (*n* = 8). Different letters indicate statistically significant differences (*p* < 0.05). **(J–N)** Representative confocal images (maximal projection) at increasing magnifications illustrating the immunolabeling of MHC class II in the DVC, co-labeled with IBA1 (microglial marker, green, *n* = 4). AP, area postrema; cc, central canal; cpNTS, commissural/postremal nucleus tractus solitarius; cmNTS, centromedial NTS; lNTS, lateral NTS; X, dorsal motor nucleus of the vagus nerve; XII, hypoglossal nucleus. IBA1, Ionized Calcium-Binding Adaptor Molecule 1.

### 3.4 CD68 expression in DVC microglia

Finally, we examined the expression of CD68, a lysosomal membrane glycoprotein implicated in endocytosis and phagolysosomal trafficking in monocytes and macrophage-lineage cells (Chistiakov et al., 2017). CD68 was strongly and mainly expressed within the DVC, more intensely in the AP, cpNTS and cmNTS, with weaker expression in the DMNX and lNTS (Figures 4A,B). This labeling co-localized with IBA1^+^ microglial cells (Figures 4C–G), although IBA1^−^ perivascular cells were also CD68-positive (Figure 4C), which is consistent with the presence of perivascular macrophages at these sites.

**Figure 4:**
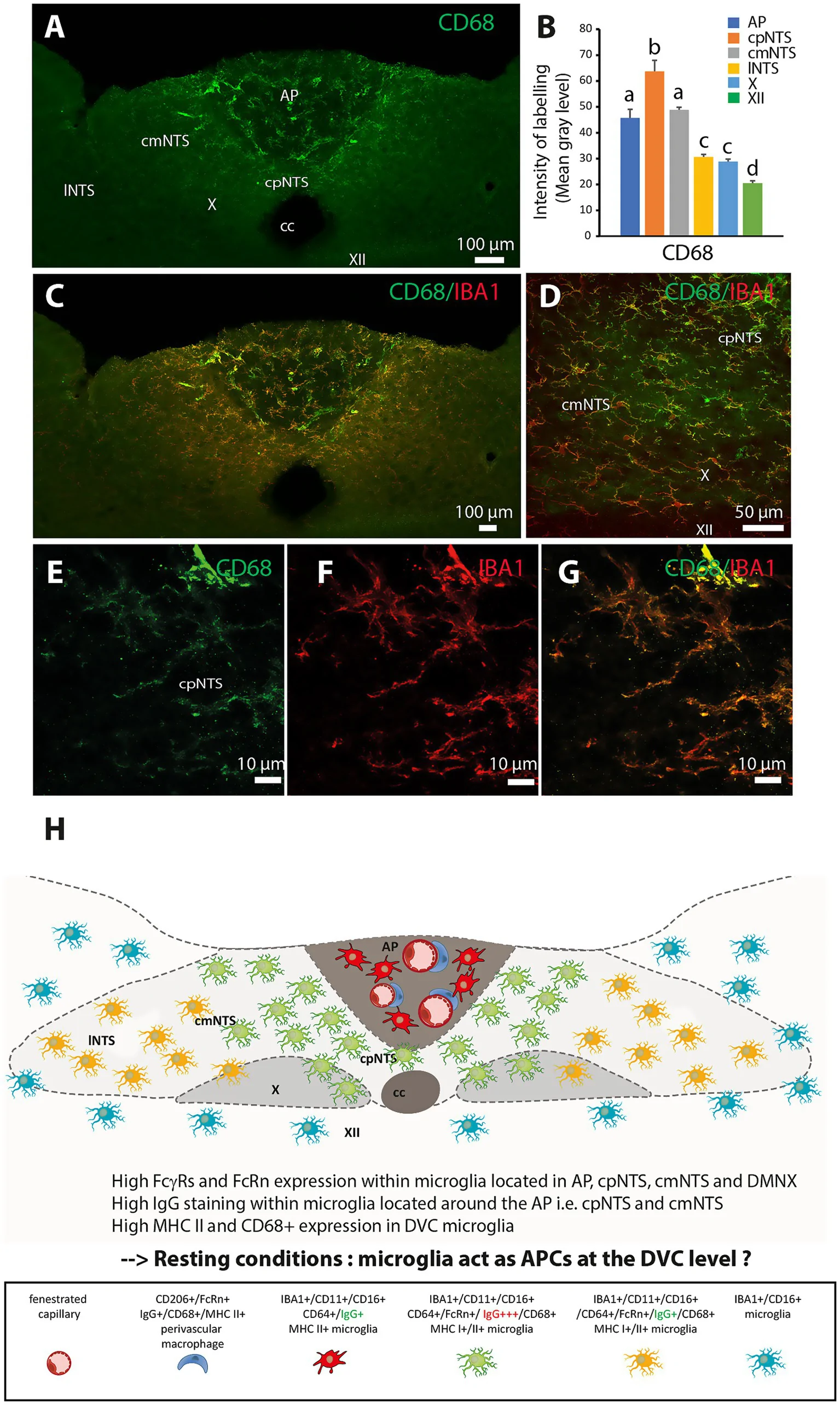
Constitutive expression of CD68 by microglial cells in the DVC. **(A)** Representative confocal image of the DVC illustrating the immunolabeling of CD68, a lysosomal membrane glycoprotein associated with endocytosis and phagolysosomal trafficking in macrophage-lineage cells. CD68 immunoreactivity is concentrated in the AP, cpNTS and cmNTS. **(B)** Histogram showing the intensity of CD68 labeling in DVC subregions and hypoglossal nucleus (*n* = 8). Different letters indicate statistically significant differences (*p* < 0.05) **(C–G)** Representative confocal images (maximal projection) at increasing magnifications illustrating the immunolabeling the CD68 and IBA1 co-labeling. CD68 co-localizes mainly with IBA1^+^ microglial cells but CD68^+^/IBA1^−^ perivascular cells are also observed (*n* = 4 per co-labeling condition). **(H)** Summary diagram of the regional distribution and molecular heterogeneity of microglial phenotypes in DVC subregions relative to surrounding neural structures. AP, area postrema; cc, central canal; cpNTS, commissural/postremal nucleus tractus solitarius; cmNTS, centromedial NTS; lNTS, lateral NTS; X, dorsal motor nucleus of the vagus nerve; XII, hypoglossal nucleus. IBA1, Ionized Calcium-Binding Adaptor Molecule 1; CD68, cluster of differentiation 68.

## 4 Discussion

In the present study, we demonstrate that microglial cells residing in the DVC constitutively express multiple FcγRs alongside MHC class I and II molecules under physiological conditions in the healthy mouse brain. Strikingly, this immunological specialization is accompanied by a selective accumulation of IgG within the DVC parenchyma, a finding consistent with the local fenestrated vasculature that unlike the canonical blood–brain barrier permits the passage of circulating plasma proteins into the tissue. Membrane receptors FcγR bind the Fc fragment of circulating IgG or immune complexes (ICs) and mediate phagocytosis and immune activation *via* the MHC class I and II molecules, which present peptide fragments derived from pathogens or endogenous proteins to cytotoxic CD8^+^ and CD4^+^ T lymphocytes, respectively, thereby bridging innate and adaptive immunity within the CNS. The co-expression of the endosomal receptor FcRn and the lysosomal marker CD68 further implies potential intracellular IgG trafficking and antigen degradation in these cells. Taken together, our results suggest that DVC microglial cells adopt an atypical phenotype in the steady-state brain, which would enable them to function as antigen-presenting cells (APCs) through a coherent and functionally integrated IgG → FcγRs → FcRn/CD68 → MHC I/II signaling axis. This possible constitutive APC capacity, operating outside of classical neuroinflammatory contexts, may represent a previously underestimated immunosurveillance mechanism unique to blood–brain barrier-deficient brain regions.

DVC microglial cells exhibit an immunophenotype radically distinct from that of their counterparts in canonical brain structures such as the cortex or hippocampus. Under basal physiological conditions, in the absence of any inflammatory stimulus or tissue injury, they constitutively express high levels of FcγRI, FcγRIII, FcRn, CD68 and both MHC class I and II molecules. This profile contrasts sharply with to the established consensus in the field. In the healthy brain, parenchymal microglia are widely reported to maintain an inhibitor-dominant expression pattern in which FcγRIIb is the dominant subtype, while activating FcγRs (FcγRI, FcγRIII) are absent or detectable only at trace levels (Fuller et al., 2014; Okun et al., 2010). This tonic inhibitory bias is thought to preserve microglial vigilance while preventing inappropriate pro-inflammatory responses to the minute amounts of IgG that cross the blood–brain barrier. It is only in pathological contexts, neuroinflammation, multiple sclerosis, Alzheimer’s disease, or viral infection, that FcγRs activation has been shown to be strongly upregulated (Ulvestad et al., 1994; van der Poel et al., 2020; Casanova et al., 2026). DVC microglia appear to constitute a conspicuous exception to this rule: under steady-state conditions, they constitutively adopt an immunophenotype typically observed only during pathological microglial stimulation. The constitutive expression of FcγRI, a high-affinity receptor for monomeric IgG, and FcγRIII, a low-affinity receptor for ICs, on AP and NTS microglial cells positions them as first-line immune sentinels, capable of binding and phagocytosing opsonized pathogens entering the DVC *via* the fenestrated capillaries of the AP. The observation of IgG associated with microglial plasma membranes, and occasionally in the cytosol, particularly in NTS cells, is consistent with a possible IgG uptake by these cells.

The origin of this parenchymal IgG, however, remains to be formally established. Although neurons have been proposed as a potential endogenous source of brain IgG (Zhang et al., 2013), we detected no IgG labeling associated with neurons in the DVC. The most parsimonious explanation remains the direct passage of circulating plasma IgG through the fenestrated capillaries of the AP, bypassing the BBB, or transcellular transport via FcRn-mediated transcytosis, IgG being by far the most abundant immunoglobulin in the bloodstream. The significant FcRn expression that we observed in AP blood vessels strongly supports this route. FcRn (encoded by *fcgrt*, also known as the Brambell receptor) is an MHC class I-like molecule associated with β2-microglobulin that resides in endosomal compartments, where it rescues pinocytosed IgG from lysosomal degradation by redirecting it back to the circulation (Schlachetzki et al., 2002; Kuo and Aveson, 2011). Highly expressed in cerebral microvascular endothelium and choroid plexus epithelium, FcRn acts as a bidirectional, homeostatic regulator of IgG concentrations at the blood–brain interface, a function particularly active in circumventricular organs (Dalakas and Spaeth, 2021; Lafrance-Vanasse et al., 2025). Remarkably, FcRn expression was detected in all microglial cells of the NTS and DMNX, but was absent in the AP. Beyond its canonical role in IgG recycling and half-life extension, FcRn has been shown to participate directly in antigen presentation (Qiao et al., 2008): distributed throughout the endolysosomal system of APCs, it facilitates the pH-dependent trafficking of IgG-complexed antigens toward MHC class II processing compartments and promotes cross-presentation on MHC class I, while unbound IgG is simultaneously recycled, a mechanism demonstrated in dendritic cells (Haddad et al., 2023; Macri et al., 2024). FcRn thus functions downstream of cell-surface FcγRs as an intracellular checkpoint that couples IgG internalization to antigen processing. Whether this mechanism operates in NTS and DMNX microglial cells remains to be tested, but is strongly suggested by our concomitant observation of CD68, a lysosomal marker of phagocytic activity, together with MHC class I and II in these same cells. The constitutive MHC II expression we observed in DVC microglia is remarkable in itself, given that MHC II levels in parenchymal microglia are otherwise negligible under homeostatic conditions (Wolf et al., 2018). In the canonical APC pathway, exogenous antigens internalized via FcγR-mediated endocytosis are processed in endo-lysosomes and loaded onto MHC II for presentation to CD4^+^ T cells, while cytosolic antigens, including proteasome-derived self-peptides, are presented to MHC I to CD8^+^ cytotoxic T lymphocytes. The co-expression of MHC class I and II molecules with the entire upstream machinery (FcγRI/III, FcRn, CD68) in DVC microglia suggests that these cells are equipped to activate both arms of the adaptive immune response.

A legitimate question arises from these observations: is the absence of a blood–brain barrier the main factor behind the unusual microglial phenotype in the DVC? As a circumventricular organ lacking a BBB, the AP is directly and continuously exposed to blood plasma constituents and blood-borne pathogens. This fundamental architectural feature may be the key determinant of local microglial identity. The fenestrated capillaries allow circulating plasma IgG (~10 mg/mL; Katsinelos et al., 2019) to freely enter the AP parenchyma, a concentration orders of magnitude higher than that found in cerebrospinal fluid (~20–30 μg/mL) or cerebral interstitial fluid (~0.1–1 μg/mL; Hirohata et al., 1984; St-Amour et al., 2013). This chronic, high-level IgG exposure may constitute the primary stimulus driving constitutive FcγR expression in AP microglia. Scattered but convergent evidence in the literature supports this interpretation. Pedersen et al. (1997) provided an early immunohistochemical characterization of immune-associated antigen expression in the AP, identifying a subpopulation of microglial/macrophage-like cells that constitutively express MHC class II, the leukocyte common antigen CD45, CD4, and ED1 under physiological conditions. More recently, Takagi et al. (2019) demonstrated that microglial cells across all CVOs of adult mice co-express CX3CR1 with low-affinity FcγRII/III and CD86, a constitutive and physiological M1-like phenotype, requiring neither infectious challenge nor inflammatory stimulation. However, our observations extend this picture beyond the AP itself. Microglial cells of the NTS and DMNX, structures located farther from the fenestrated capillaries but still within the DVC, also constitutively express FcγRs, FcRn, as well as MHC class I and II molecules. This indicates that the atypical microglial phenotype is not limited to the BBB-deficient zone of the AP but spreads to adjacent integrative and motor nuclei, with potentially broad consequences for autonomic information processing. Furthermore, a more detailed comparison of microglial phenotypes across DVC subregions reveals meaningful molecular heterogeneity: FcRn and MHC class I are expressed by microglia in the NTS and DMNX but are obviously absent from AP microglia. This differential expression pattern suggests that, far from being uniform, the immunological specialization of DVC microglia is regionally tuned, with distinct functional repertoires that may reflect the specific physiological demands of each nucleus (Figure 4H).

Taken together, our results delineate a coherent IgG → FcγRI/III → FcRn/CD68 → MHC I/II signaling axis constitutively expressed by discrete subpopulations of microglial cells across multiple DVC subregions under physiological conditions, without an apparent requirement for inflammatory priming. This constitutive specialization appears inseparable from the DVC’s broader functional identity as a privileged hub for the integration and relay of peripheral inflammatory signals to the CNS. Emerging evidence underscores the functional importance of DVC microglia in this context. Castellanos et al. (2026) recently showed that intra-fourth-ventricle administration of minocycline, which reduced IBA-1 immunoreactivity in the AP and NTS, significantly attenuated c-Fos induction in the DVC following intraperitoneal TNFα challenge.

These results suggest that resident microglia are active participants, not passive bystanders, in the brainstem’s response to peripheral pro-inflammatory signals, and that their depletion selectively disrupts autonomic circuitry activation. Complementary evidence comes from Gaige et al. (2022), who demonstrated that microglial depletion via PLX3397 paradoxically exacerbated neuronal activation in the DVC induced by deoxynivalenol, a mycotoxin of dietary origin with neuroinflammatory properties. This gain-of-activation phenotype upon microglial loss strongly suggests that DVC microglia normally exert a neuroprotective, dampening influence on the parenchymal response to blood-borne hazardous molecules. The constitutive expression of the FcγR/IgG/MHC class I and II pathway in DVC microglial cells could thus represent a molecularly defined substrate for this heightened surveillance capacity. This pathway potentially functions as a permanent immunological interface at the blood–brain boundary, with possible consequences for the transduction of peripheral immune information into autonomic, behavioral, and physiological responses.

## Data Availability

The raw data supporting the conclusions of this article will be made available by the authors, without undue reservation.
